# COMPLEXITY IN PSYCHOSOCIAL INTERVENTIONS: CASE STUDIES FROM A STROKE TRANSITIONS TRIAL

**DOI:** 10.1093/geroni/igac059.1538

**Published:** 2022-12-20

**Authors:** Emmanuel Chima, Amanda Woodward, Anne Hughes, Michele Fritz, Paul Freddolino, Sarah Swierenga, Constantinos Coursaris, Mathew Reeves

**Affiliations:** Michigan State University, East Lansing, Michigan, United States; Michigan State University, Ann Arbor, Michigan, United States; Michigan State University, East Lansing, Michigan, United States; Michigan State University, East Lansing, Michigan, United States; Michigan State University, East Lansing, Michigan, United States; Michigan State University, East Lansing, Michigan, United States; HEC Montreal, Montreal, Quebec, Canada; Michigan State University, East Lansing, Michigan, United States

## Abstract

The Michigan Stroke Transitions Trial (MISTT) tested whether in-home social work case management (SWCM) or SWCM combined with access to a website providing stoke-related information improved outcomes relative to usual care for patients discharged home post-stroke and their caregivers. The aims of this secondary analysis are 1) to describe the actual support social work case managers (SWCM) provided to MISTT participants and 2) use select case studies to illustrate the relationship between SWCM and quantitative patient and caregiver outcomes. Data for the study were derived from SWCM case notes on 157 patients and their caregivers who received the MISTT intervention. Case notes were coded in two steps with a subset of cases coded by two researchers and reviewed for interrater reliability in each step. The first round of coding was guided by primary SWCM intervention goals. The second round of coding identified SWCM sub-themes within each primary goal. Key themes indicate SWCMs aided with understanding the post-hospitalization period, helped patients navigate a range of systems and services, identified needs and supported patient goals, provided psychosocial support, and centered support on stroke recovery and prevention. Case studies illustrate ways in which SWCM were key supports during the transition period, but that support does not cleanly align with quantitative findings from patient-reported outcomes. This study aligns with a growing body of work documenting the complexity of transitions of care and has implications for how we support patients and caregivers as they move from inpatient to outpatient care and measure outcomes.

